# PANCREATIC CANCER PROLIFERATION IS INDUCED BY GLI2-COL1 SIGNALING IN FIBROBLASTS IN THE TUMOR MICROENVIRONMENT

**DOI:** 10.1093/geroni/igac059.2837

**Published:** 2022-12-20

**Authors:** Madeline Ross, Renzo Vera, Maite Fernandez-Barrena, Murat Toruner, Ezequiel Tolosa, Luciana Almada, Rolf Brekken, Martin Fernandez-Zapico

**Affiliations:** St. Catherine University, St. Paul, Minnesota, United States; Mayo Clinic Schulze Center for Novel Therapeutics, Rochester, Minnesota, United States; Mayo Clinic Schulze Center for Novel Therapeutics, Rochester, Minnesota, United States; Mayo Clinic Schulze Center for Novel Therapeutics, Rochester, Minnesota, United States; Mayo Clinic Schulze Center for Novel Therapeutics, Rochester, Minnesota, United States; Mayo Clinic Schulze Center for Novel Therapeutics, Rochester, Minnesota, United States; UT Southwestern, Dallas, Texas, United States; Mayo Clinic Schulze Center for Novel Therapeutics, Rochester, Minnesota, United States

## Abstract

Pancreatic ductal adenocarcinoma (PDAC) affects many older adults in the United States. This aggressive type of pancreatic cancer is resistant to many chemotherapies, resulting in a poor prognosis and a five-year survival rate of less than ten percent. To study PDAC progression and develop novel therapies, it is important to better understand the tumor microenvironment (TME) and the contributing cell populations and molecular mechanisms. Despite the well-described role of soluble Type I collagen in the regulation of pancreatic cancer TME, the molecular events modulating its expression/activity remain elusive. Here, we describe a novel mechanism controlling the levels of soluble collagen in cancer associated fibroblasts (CAFs), a major producer of Type I collagen in pancreatic cancer TME. Specifically, we provide evidence that the transcription factor GLI2 is required for the expression of COL1A1, a key component of the Type I collagen fiber, stimulated by the transforming growth factor β (TGFβ). Our methodology included expression studies from patient samples, ChIP-qPCR, qPCR, RNA-seq analysis, gene set enrichment analysis, biological assays, and gene ontology analysis. Our results confirm that the TGFβ-GLI2 axis in CAFs is able to activate EGR1-proliferative signaling downstream of collagen signaling in pancreatic cancer cells contributing to our understanding of the molecular underpinnings of pancreatic cancer TME. Further studies that define the complete role of EGR1 in PDAC may lead to the development of novel therapies targeting EGR1 or CAFs to promote enhanced quality of life following a PDAC diagnosis.

